# Molecular, Structural, and Rheological Characterization of Camel Skin Gelatin Extracted Using Different Pretreatment Conditions

**DOI:** 10.3390/foods10071563

**Published:** 2021-07-06

**Authors:** Olumide Samson Fawale, Ahlam Abuibaid, Fathalla Hamed, Phanat Kittiphattanabawon, Sajid Maqsood

**Affiliations:** 1 Department of Food Science, College of Food and Agriculture, United Arab Emirates University, Al Ain 15551, United Arab Emirates; 201990012@uaeu.ac.ae (O.S.F.); Ahlam_k@uaeu.ac.ae (A.A.); 2 Department of Physics, College of Science, United Arab Emirates University, Al Ain 15551, United Arab Emirates; fhamed@uaeu.ac.ae; 3 Department of Food Science and Technology, Faculty of Agro and Bio Industry, Thaksin University, Phatthalung Campus, Phatthalung 93210, Thailand; phanat@tsu.ac.th

**Keywords:** camel skin gelatin, characterization, gel strength, FTIR, XRD

## Abstract

Optimum conditions for high-quality gelatin recovery from camel skin and its molecular, structural, and rheological characterization were carried out in this study. Increased yield and gel strength were recorded, with an increase in camel skin pretreatment times of 6 to 42 h and 0.50 and 0.75 M-NaOH. Gelatin from skin pretreated with 0.75 and 0.5 M-NaOH for 42 h showed the highest yield (22.60%) and gel strength (365.5 g), respectively. Structural characterization by Fourier transformation infrared spectra, X-ray diffraction, and nuclear magnetic resonance indicated that all gelatins possessed major peaks in the amide region, and diffraction peaks around 22° were basically amorphous. The temperatures for gelling and melting ranged from 20.9 °C to 25.8 °C and 27.34 °C to 30.49 °C. Microstructure revealed loose network with more voids in gelatin from skin pretreated with 0.5 and 0.75 M-NaOH for 6 h, while a highly cross-linked network and less voids were observed in those pretreated with 0.75 M-NaOH for 24 h and 0.5 M-NaOH for 42 h. The results reveal that great potential exists in producing halal gelatin with excellent quality and functionality from camel skin.

## 1. Introduction

Gelatin is an essential protein present in the skin, bones, and connective tissues of animals, formed by partial hydrolysis of collagen [1]. It possesses unique properties, such as the formation of thermo-reversible gels close to body temperature and is readily soluble in water. Gelatin is regarded as a special hydrocolloid that can be utilized in the food, pharmaceutical, and cosmetic industries [2]. Researchers have shown that commercially manufactured gelatin is primarily extracted from porcine skin (46%), bovine bones (28.5%), and bovine hides (29.5%). However, some concerns exist for these sources, such as mad cow disease from bovine sources and religious restrictions which exist for the porcine products, which have affected the gelatin market in the past. Despite these concerns, in 2011, a significant increase in the global estimated usage of gelatin was reported (348.9 thousand tons). Moreover, prior to COVID-19, global projections predicted a significant rise to about 450.7 thousand tons by 2020 [3]. The rapid growth in the international market for halal-certified foods has prompted food technologists to look beyond the traditional sources of gelatin for an alternative source [4]. However, there are few halal alternative mammal sources available for commercial gelatin production on a large scale.

In the Middle East, some African countries, and many other areas, camels are raised domestically as a food source, and their skin and bones are considered potential sources of halal gelatin. The global camel population is reportedly about 35 million [5]. Camels are unique animals and are thought to possess unusual skin composition, structure, and proteins because of their daily exposure to dry and hot climatic conditions. The skin is a major by-product of slaughtering camels (10–15% of body weight) and a potential source of raw material for gelatin extraction. Furthermore, a significant increase in the annual number of camels slaughtered in the United Arab Emirates (UAE) (72,606 to 168,000) occurred between 2000 and 2013, and the number of camels slaughtered globally increased from 1,672,870 in 2000 to 2,568,266 in 2013 [5], which has generated vast quantities of by-products (including the skin). Thus, the potential of these products has not yet been fully elucidated and these may be a novel source to produce BSE-free alternative products such as high-quality gelatin. To date, the structure of camel skin and the characterization of the collagen protein have not been studied. In a recent study by [6], gelatin was extracted from 2.5-, 4.5-, and 7-year-old camel skins, and the results revealed that the gel strength, which is an important functional property of gelatin, was quite low (72.08–122.87 g). The lower gel strength was attributed to some intrinsic properties, such as the molecular weight distribution, low protein content, and extraction procedure used. The study of [6] did not carry out optimization of the pretreatment condition of skin which has a very important contribution in recovering high amounts of gelatin with high gel strength.

Gelatin extraction from the skin of various animals is commonly accomplished using acid or alkaline pretreatments to confer desirable properties to the gelatin. The effectiveness of gelatin extraction and quality are influenced by the process in which collagens (parent protein for gelatin) are pretreated [7]. Therefore, the optimum level of pretreatment of skin during gelatin extraction plays an important role in determining the yield, gel strength, and viscosity. However, there is currently no research that focuses on optimizing camel skin pretreatment conditions to optimize both gelatin yield and gel strength. The optimization of the pretreatment condition of the skin is an extremely important step for recovering high-quality gelatin. Therefore, an in-depth study into the characterization of gelatin from camel skin using pretreatments with various levels of alkali reagents at different time intervals is required. This study sought to determine the optimal conditions for the recovery of high-quality gelatin from camel skin pretreated with various levels of sodium hydroxide at different time intervals, and to carry out an in-depth characterization of the camel skin gelatin with respect to the molecular, structural, rheological, and gelling attributes.

## 2. Materials and Methods

### 2.1. Camel Skin Preparation

Fresh skin was collected from a nearby slaughterhouse in Al Ain, United Arab Emirates, from healthy camels slaughtered at 3 years of age and transported directly to the laboratory. Upon arrival in the laboratory, the skin was washed thoroughly with distilled water and visible meat was removed. The skin was cut into small parts (3 × 3 cm^2^) and frozen at −20 °C for 2 weeks.

### 2.2. Alkaline Pretreatment of the Camel Skin and Extraction of Gelatin

**Figure 1:**
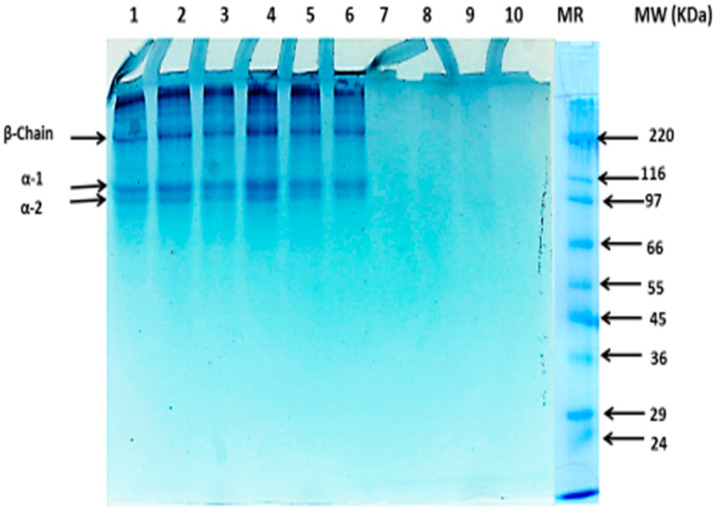
Protein pattern of gelatin from camel skin pretreated with different alkaline concentration for various times. Keynotes: MR denotes high molecular weight protein markers. Lane 1: 0.5 M-6 h; Lane 2: 0.75 M-6 h; Lane 3: 0.5 M-12 h; Lane 4: 0.75 M-12 h; Lane 5: 0.5 M-24 h; Lane 6: 0.75 M-24 h; Lane 7: 0.5 M-2D; Lane 8: 1.M-2D; Lane 9: 0.5 M-4D; Lane 10: 1.0 M-4D.

To optimize the pretreatment conditions, a preliminary study was conducted, in which the skin samples were soaked in 0.5 and 1.0 M NaOH with a skin/solution ratio of 1:10 (*w*/*v*) for 2 and 4 days. It was found that the gelatin obtained from the preliminary study was not able to form a gel and all the protein bands were degraded (Figure 1). Thus, the main experiment was conducted using lighter pretreatment conditions (0.5 M and 0.75 M NaOH) and soaking was carried in 6 h intervals. The skin was further treated with 0.5 and 0.75 M NaOH at 1:10 (*w*/*v*) skin/solution for 6, 12, 24, 30, 36, and 42 h at 28–30 C, and the mixture was manually stirred twice daily. With deionized water, the alkaline-pretreated skins were cleaned several times until neutral pH was attained. At 70 °C, the washed and neutralized skins were extracted for 5 h at a speed of 150 rpm in a water bath (Memmert, Schwabach, Germany) connected with an overhead stirrer equipped with a propeller (Scientific Chemical Technologies, Boston, MA, USA) set. Following the extraction, 2 layers of cheesecloth were used to filter the solution, and the obtained filtrates were then filtered through Whatman No. 4 filter paper under vacuum using a Buchner funnel. The filtrates were lyophilized using a freeze dryer (−81 °C, 0.044 mBar) (Telstar, Woerden, The Netherlands) and were referred to as 0.5 M-6 h, 0.5 M-12 h, 0.5 M-24 h, 0.5 M-30 h, 0.5 M-36 h, and 0.5 M-42 h for 0.5 M NaOH pretreated samples and 0.75 M-6 h, 0.75 M-12 h, 0.75 M-24 h, 0.75 M-30 h, 0.75 M-36 h, and 0.75 M-42 h for 0.75 M NaOH pretreated samples. The extracted gelatin powder samples were analyzed in triplicate, as described hereafter.

### 2.3. Analyses

#### 2.3.1. Yield of Extraction

The gelatin powder produced following freeze-drying was weighed, and the yield was calculated using this equation [8]:

$$\mathrm{Yield}\;\left(\%\right)=\frac{\mathrm{weight}\;\mathrm{of}\;\mathrm{dried}\;\mathrm{gelatin}\;\left(g\right)}{\mathrm{weight}\;\mathrm{of}\;\mathrm{initial}\;\mathrm{fresh}\;\mathrm{skin}\;\left(g\right)}\times 100^{}$$

The proximate composition of the camel skin was evaluated as shown in Supplementary Materials.

#### 2.3.2. Determination of Gel Strength

In cylindrical molds, (diameter = 3 cm × height = 2.5 cm), gelatin gels (6.67%, *w*/*v*) were prepared using the method of [9]. The gel strength was determined using a texture analyzer (CT3-4500, Brookfield Engineering Laboratories, Middleboro, MA, USA) equipped with a 5 kg load cell with a flat-faced cylindrical teflon plunger (diameter 1.27 cm). In terms of the maximum force in grams necessary for the plunger to penetrate the gelatin gels at 4 mm, the gel strength was determined.

#### 2.3.3. Sodium Dodecyl Sulfate–Polyacrylamide Gel Electrophoresis (SDS-PAGE)

Camel skin gelatin SDS-PAGE was conducted according to a method defined previously by [10]. Briefly, the gelatin samples (15 μg protein) were analyzed through electrophoresis by loading into polyacrylamide gels containing 4% of stacking and 7.5% running gel at a constant current of 20 mA/gel. Pre-stained protein standards were used with a broad molecular weight range (10–250 kDa).

### 2.4. Structural Characterization of the Camel Skin Gelatin

#### 2.4.1. Fourier Transform Infrared Spectroscopy Analysis (FTIR)

The FTIR spectra of the gelatin samples were determined by the method described by [10]. A deuterated L-alanine triglycine sulfate detector attached with the FTIR spectrometer (Nicolet, Thermo Electron, Waltham, MA, USA) was used for this study, and spectra obtained at a resolution of 4 cm^−1^ using a range of 4000–650 cm^−1^ (mid-IR region).

#### 2.4.2. Proton Nuclear Magnetic Resonance (1H NMR)

The 1H NMR analysis was performed on the camel skin gelatin (0.75 M-42 h) samples prepared using JEOL ECA-600 spectrometer at a concentration of 10 mg/mL in D_2_O (1H 600 MHz, 13C 150 MHz). The gelatin solution was transferred to the NMR tubes (5 mm in diameter), and the spectra were measured with MestReNova software. Using the methods described by [11], all the chemical shifts were reported in parts per million (ppm).

#### 2.4.3. X-ray Diffraction Determination (XRD)

Gelatin crystal structures were determined from the powder XRD measurements using a Bruker D2 Phaser Powder X-ray diffractometer with CuKa radiation in a 2θ range of 10–80° with a step mode of 0.02°/min.

#### 2.4.4. Microstructural Features by Scanning Electron Microscopy

For the microstructure of the gelatin gels, scanning electron microscopy (SEM) (JEOL JSM-5800 LV, Tokyo, Japan) at an acceleration voltage of 20 kV was used according to the method earlier described by [10].

### 2.5. Determination of the Gelling and Melting Temperature

For melting and gelling temperature profile, a controlled stress rheometer (Hybrid Rheometer, TA Instrument, New Castle, DE, USA) was used according to the method described by [12].

The techno-functional properties (solubility, emulsifying, and foaming properties), as well as turbidity, were determined, as described in Supplementary Materials.

### 2.6. Statistical Analysis

Camel skin pretreatment using 2 NaOH levels and separate soaking time intervals was performed in 3 batches. All analyses were done in triplicate and statistical Package for Social Sciences (SPSS for windows: SPSS Inc., Chicago, IL, USA) was used in analyzing the data and mean comparisons were made using Duncan multiple range test.

## 3. Results and Discussion

### 3.1. Yield of Extraction

Extracted gelatin yield in this study varied depending on the concentration of the alkaline solution used and the duration of the pretreatment. As depicted in Table 1, an increase in the time and concentration of the NaOH pretreatment gave a corresponding increase in the yield of gelatin. The highest extraction yield was observed with skin pretreated at 0.75 M-42 h (22.6%), which was significantly higher (*p* < 0.05) compared to the yield obtained using other pretreatment conditions. In the skin matrix, repulsion in the protein chains were more pronounced, leading to an increase in solubility resulting in higher extraction of the gelatin from camel skin treated for a longer period of time (42 h) with higher NaOH concentrations. However, for easy destabilization of cross-links in the skin matrix, which will give rise to lower cross-links in the collagen molecule, pretreating with a weak alkaline concentration for a lesser time might not be enough. Lower crosslinking in the collagen molecule, connective tissues, and protein compositions might be responsible for a lower yield of gelatin using some of the pretreatment conditions [13]. The yield of camel skin gelatin in this study was affected by the NaOH concentration and was higher compared to the gelatin from the alkaline-pretreated goat skin (15.95%) at 0.75 M NaOH for 2 days [10], higher than the bullfrog gelatin yield (15.40%), and frog gelatin yield reported by [14,15]. Al-Hassan [6] recorded higher gelatin yields of 36.8%, 37.4%, and 42.4% from 2.5-, 4.5-, and 7-year-old camel skins pretreated with calcium hydroxide [Ca(OH)_2_] for 48 h, which might be due to the fact that the extraction procedure used in this study was different from that used in the present study.

For instance, the extraction time and temperature used were different, the skin was soaked in calcium hydroxide, and filtration was not conducted by [6]. In addition, in prior research by [16] on camel bone gelatin, the yields ranged from 8.54% to 25.33% when extraction conditions were applied at temperature (40, 60 and 80 °C), time (0.5, 2.0 and 3.5 min), and pH (1, 4 and 7). Research has shown that the extraction yield of gelatin is affected by several factors, such as the tissue type as well as the pretreatment and extraction conditions.

### 3.2. Gel Strength

As shown in Table 1, overall, there was an increase in the gelatin gel strength when NaOH concentration and the time used for the pretreatment were increased. The highest gel strength (365.5 g) was recorded for the gelatin obtained from the pretreated camel skin at 0.5 M NaOH for 42 h (*p* < 0.05), which was higher than the gel strength previously reported for the gelatin (120.56, 122.87, and 72.08 g) from 2.5-, 4.5-, and 7-year-old camel skins [6], commercial bovine gelatin (293.22 g) [7], Asian bullfrog (*Rana tigerina*) skin (between 130 and 248 g) [14], and goat skin (209.18 to 229.52 g) [10]. The lowest gel strength of gelatin (175 g) was reported for the gelatin obtained from the camel skin pretreated using 0.5 M NaOH for 6 h, which was, in fact, higher than the previously reported gel strength for camel skin gelatin [6] and unicorn leather jacket fish skin (149.77 g) pretreated with different acids [7]. Benjakul et al. [17] reported that the chemical composition of gelatin is greatly affected by the raw material and pretreatment conditions, which, in turn, affected the functional properties, particularly the gel strength. Variations in the proline and hydroxyproline content, as a result of the differences in the raw material, were factors that influenced the gelling properties of gelatin and the mammalian gelatin that reportedly exhibited higher amounts of hydroxyproline and proline than the fish gelatins [13]. Higher molecular weight gelatin polypeptides, obtained because of longer pretreatment times, exhibited a higher gel strength than that of the low molecular weight peptides because of their inability to form inter-junction zones [18]. The gel strength of the commercial bovine and porcine gelatins was reportedly in the range of 100 to 300 g, but 250–300 g of the bloom strength was highly desirable for food and pharmaceutical applications. Therefore, the highest gel strength (365.5 g) reported in this study was higher compared to the previously reported bovine, goat, and fish skin gelatin, which demonstrated potential as a BSE-free, halal-certified alternative to conventional gelatin intended for food and pharmaceutical applications.

### 3.3. Protein Pattern of the Camel Skin Gelatin as Affected by the Alkaline Pretreatment

The protein pattern of gelatin from camel skin pretreated using varying concentrations of NaOH at various soaking times is presented in Figure 1. The molecular distribution pattern revealed that all the samples had α- and β-chains, except for gelatin obtained from the preliminary experiment. The intensity of the polypeptide chains of the extracted gelatins was affected by the pretreatment condition of the skin and time of exposure.

The gelatin samples obtained in the preliminary experiment displayed complete degradation of all the proteins, as can be seen from the SDS-PAGE gel image (Lane 7–10). Al-Hassan [6] also reported that all of the proteins in the gelatin extracted from the 7-year-old camel skins pretreated with Ca(OH)_2_ for 48 h underwent complete degradation, and [19] reported that α-chains were more degraded in the channel catfish skin gelatin pretreated with 1 g/L (Ca(OH)_2_) for 76 h. However, camel skin pretreated with 0.5 and 0.75 M NaOH for 6, 12, and 24 h (Lane 1 to 6, Figure 1) in this study revealed that the resultant gelatin samples exhibited intact α_1_-, α_2_-, and β-chains. The β-chain had a molecular weight (MW) of 225 kDa, and the α-chain was composed of α_1_- and α_2_-chains with estimated molecular weights of 120 and 116 kDa, respectively. The protein patterns among the gelatin obtained from the camel skin pretreated with different concentrations of NaOH (0.5 and 0.75 M) for up to 24 h showed no noticeable differences. The results revealed that the pretreatment process conducted for 24 h did not degrade α- and β-chains in the gelatin. Mad-Ali et al. [10] reported comparable results in the protein pattern of the goat skin gelatin with varying alkaline pretreatment conditions, and no noticeable differences were observed by varying the alkaline pretreatment conditions. Retaining and preventing degradation of the α- and β-chains is very important because higher gelling, emulsifying, and foaming were reported in the gelatins with a higher α-chain content. The camel skin gelatin protein components (α- and β-chains) in this study were tolerant of the alkaline pretreatment until 24 h of exposure, and then they began to degrade and were completely degraded at 48 h (2 days) of the alkaline pretreatment process at NaOH concentrations of 0.5 and 1.0 M.

### 3.4. Structural Elucidation of the Camel Skin Gelatin via Fourier Transform Infrared (FTIR) Spectroscopy

**Figure 2:**
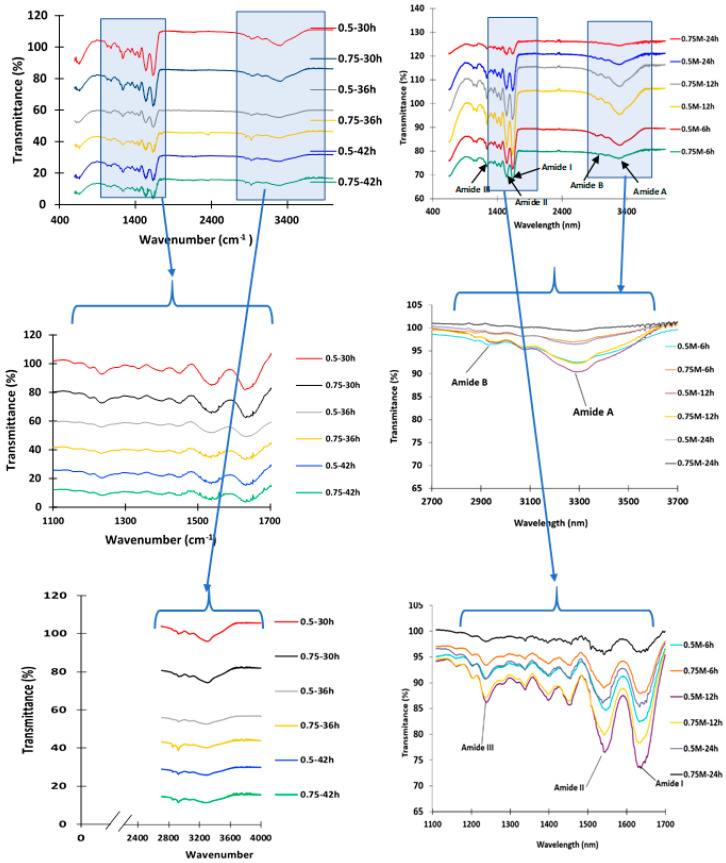
Fourier transform infrared (FTIR) spectra of gelatin from camel skin pretreated with 0.5 and 0.75 M NaOH for 6, 12, 24, 30, 36 and 42 h. For keynotes, please see footnotes of Table 1.

The gelatin FTIR spectra showed major peaks in the amide region (amide I, II, and III, and amide A and B) (Figure 2), and the transmittance for all the amide peaks of the gelatin from the preliminary experiment (Supplementary Figure S1) exhibited a lower intensity or disappeared completely when compared to that of the amide peaks of the gelatin samples from the main experiment (Figure 2). All the amide peaks of the gelatin extracted with 1 M NaOH for 4 days had completely disappeared, which indicated that the longer pretreatment time had adversely affected all the functional groups (Supplementary Figure S1). The gelatin samples from the preliminary experiment with a pretreatment duration of 2 and 4 days also showed degradation of the protein bands as displayed in the SDS-PAGE gel (Figure 1) and, thus, were not able to form a gel. The FTIR spectra for all the gelatin samples (0.5 M–0.75 M NaOH for 6, 12, 24, 30, 36, and 42 h pretreatment) displayed prominent amide I, II, and III, as well as amide A and B peaks. Gelatin obtained from the camel skin treated with 0.75 M NaOH for 36 and 42 h displayed spectra that were similar to those of the gelatin samples from the preliminary experiment (0.5–1.0 M NaOH for 2 days) (Supplementary Figure S1). The camel gelatin from the skin pretreated with 0.5 and 0.75 M NaOH for 6, 12, and 24 h displayed an amide I peak from the wavenumber, which ranged from 1616.8 to 1680.9 cm^−1^ (Figure 2). The amide I peak of the gelatin from the skin pretreated with 0.5 and 0.75 M NaOH for 30, 36, and 42 h appeared in the wavenumber ranging from 1635 to 1641 cm^−1^. The literature revealed that the amide I vibration mode was mainly a C=O stretching coupled with C–N stretch, CCN deformation, and N-H bending modes in the plane. Moreover, an infrared spectroscopic analysis was used in studying the secondary structure of the protein, the absorption in the amide I region, which was potentially the most useful [20], and the characteristic coil structure of the gelatin as a result of the absorption peak at amide I [9]. In all the gelatin samples, amide II bands were observed at a wavenumber that ranged from 1520.1 to 1560.0 cm^−1^ and was caused by an out-of-phase combination of the C–N group stretch vibration and N–H group distortion modes of the peptide group [9]. The amide III band was reflected as a very low intensity peak at a wavenumber ranging from 1240 to 1247 cm^−1^ for the gelatin samples, which is attributable to the disorder arrangement in the gelatin molecules and loss of the triple helix state in the gelatin [21].

The most noticeable differences found among the sample were in the peak of amide A and detected at wavelengths ranging from 3250 to 3352 cm^−1,^ which corresponds to stretching vibration of the N–H group linked with hydrogen bonding [10]. Overall, the gelatin extracted from the skin pretreated with 0.75 M NaOH for a duration longer than 24 h displayed a lower transmittance percentage for amide I, II, and A. In this study, the camel skin gelatin secondary structure and functional groups were greatly affected by the alkaline pretreatment, with the harsh alkaline treatment extended beyond 2 days showing a significant effect.

### 3.5. Structural Elucidation of the Camel Skin Gelatin by Nuclear Magnetic Resonance (NMR) Spectroscopy

**Figure 3:**
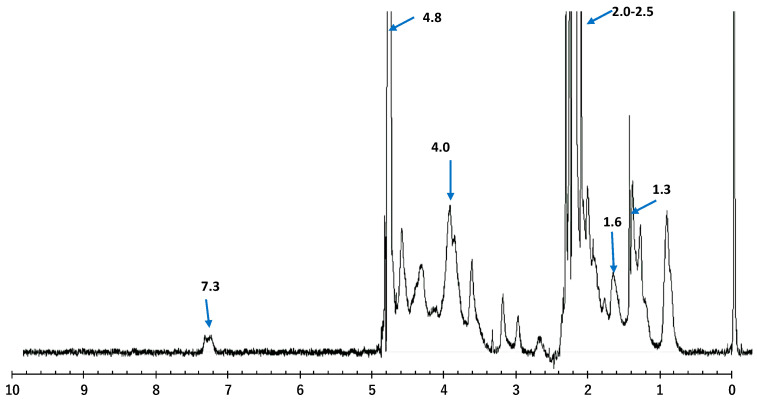
Nuclear magnetic resonance spectroscopy (1H NMR) spectra of gelatin from camel skin treated with 0.5 M NaOH for 42 h.

NMR spectroscopy is an analytical tool used in the identification of the constitution, conformation, and configuration of natural molecules. 1H-NMR has been used as an efficient tool to monitor the position of the hydrogen atoms within the molecular construction of complex structures such as proteins [22]. As depicted in Figure 3, the NMR spectra exhibited an array of peaks between 0 and 8 ppm, owing to gelatin being made up of many amino acids.

Two major intense bands of water molecules at 2.2 to 2.5 and 4.8 ppm were revealed by the NMR spectrum within the gelatin coiled structure surrounded by the amino acids, which stabilized the helical structure via the formation of hydrogen bonds between adjacent chains [22]. Uriarte-Montoya et al. [23] reported similar band spectra for giant squid skin gelatin. The peaks observed at 2.2 and 4.8 ppm indicated regions around the aliphatic protons attached to the carbon atoms (valine, leucine, and isoleucine), aliphatic carbon protons (arginine, leucine, lysine, proline, glycine, and aspartic acid), and α-CH of the amino acids. The bands detected at 1.3 and 1.6, and the prominent band at 4.0, indicated protein unfolding and the exposure of the amide regions or groups and α-carbon protons within the protein structure [24]. The resonance signal around 7.3 ppm due to the aromatic protons of phenylalanine, agreed with the report of [25] on the porcine skin gelatin. The NMR result obtained in this study was similar to the report of [26], where the characteristic signals of the fish gelatin were reported to be crowded into a narrow region between 0 and 5 ppm. Moreover, the results obtained in this study suggested that in the presence of radio waves with different frequencies, minimum cross-linking, as well as loss of order, was observed in the chain, making the amino acid less attached to the main chain. This caused them to be more prone to interaction with solvents that could lead to modifications in the chemical environment [23].

### 3.6. X-ray Diffraction (XRD) Analysis of Camel Skin Gelatin

**Figure 4:**
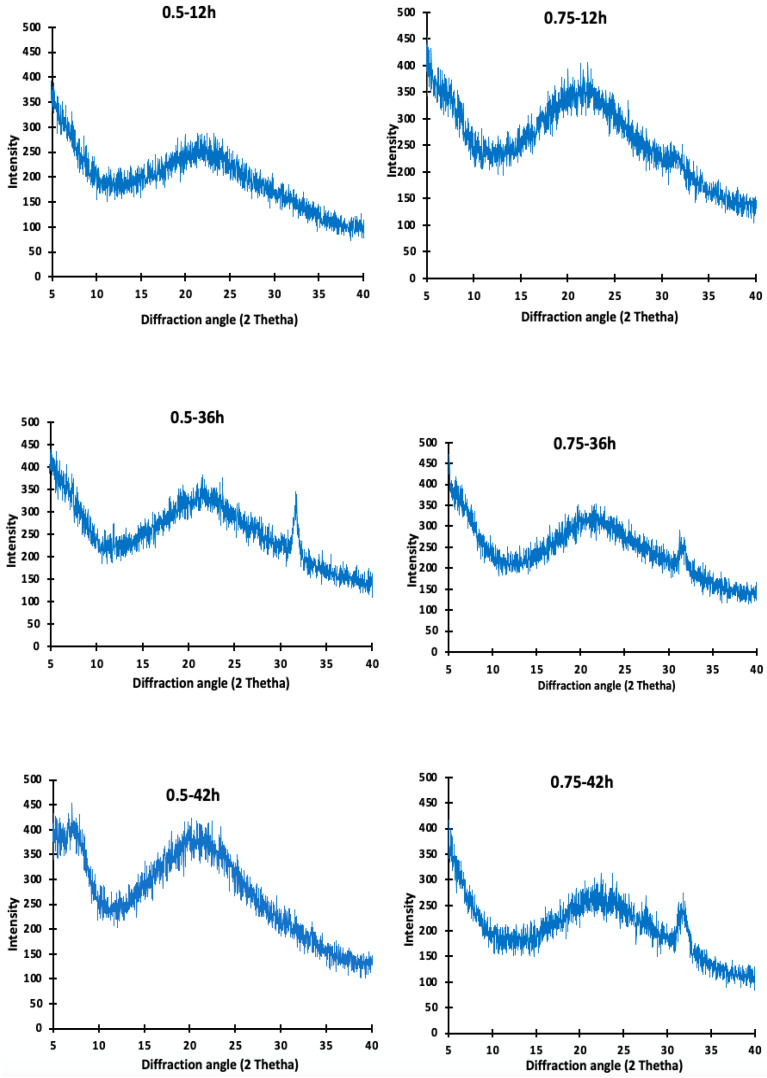
X-ray diffraction patterns of (XRD) spectra of gelatin from camel skin pretreated with 0.5 and 0.75 NaOH for 12, 36 and 42 h. Keynotes: 0.5 M-12 h; 0.75 M-12 h; 0.5 M-36 h; 0.75 M-36 h; 0.5 M-42 h; 0.75 M-42 h denotes gelatin obtained from the camel skin treated with 0.5 or 0.75 M NaOH for 12, 36, and 42 h, respectively.

The gelatin XRD patterns obtained from the camel skins pretreated with 0.5 M and 0.75 M-12 h, 0.5 M and 0.75 M-36 h, and 0.5 M and 0.75 M-42 h are depicted in Figure 4. The diffractograms observed in all of the camel skin gelatin showed a characteristic amorphous structure. No visible differences were found between the crystal structures of the camel skin gelatin obtained from the various pretreatments. A sharp diffraction peak was observed at 2θ = 7° for the gelatin extracted at 0.5 M NaOH for 42 h, and broad peaks located at 2θ = ~20–22° were observed in all the gelatin samples. A longer extraction time appeared to be linked to the destruction of the cross-link in the skin matrix, thereby promoting the recovery of the triple helix structure of the gelatin present in the collagen [27]. Badii et al. [27] had previously reported a direct correlation between gel strength and the triple helix content measured via the XRD. In the present study, the gelatin samples were extracted for an extended period of 48 h. The triple helix contents were determined via X-ray diffraction analysis and were consistent with the results of the gel strength reported in this study. However, it was observed that an increase in the extraction time led to a slight shift in the diffraction peaks of the gelatin samples, and a new characteristic peak appeared at 2θ = 32°, 32.1°, and 32.02° in the gelatin samples from the camel skin that had underwent prior treatment at 0.5 and 0.75 M NaOH for 36 h and 0.75 M NaOH for 42 h, which indicated the formation of a new crystal structure.

This shift could be the result of the variation in the gelatin origin and the moisture content of the gelatin samples [28]. It should also be noted that as the time duration of the extraction increased, the intensity of the peak also increased.

### 3.7. Microstructure of Gelatin

**Figure 5:**
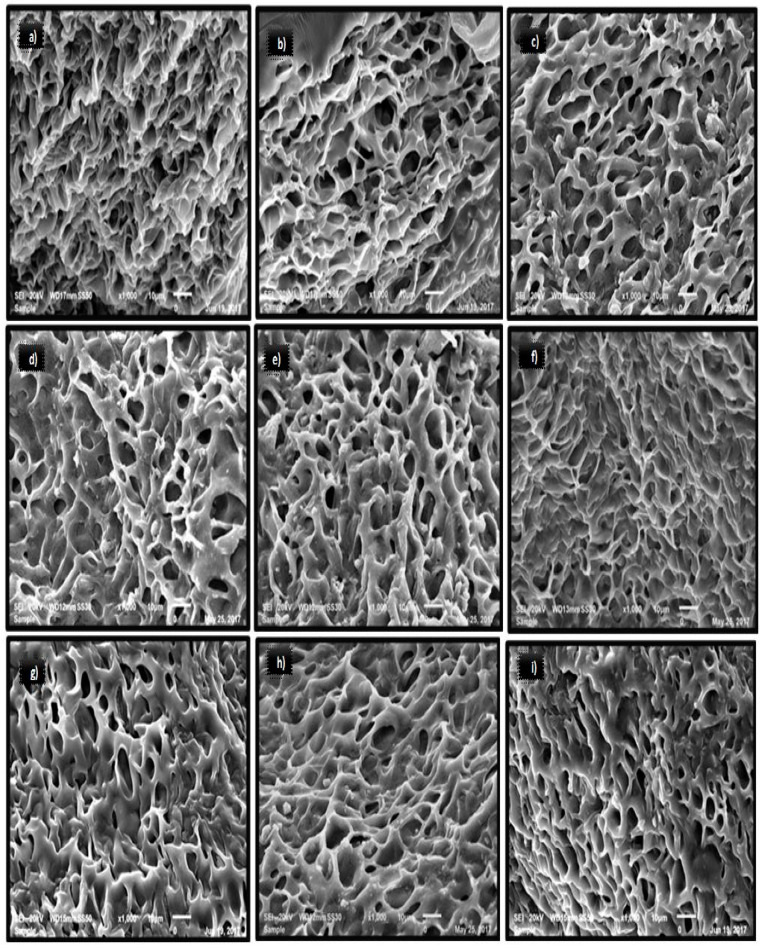
Microstructural features of gelatin from camel skin treated different concentration of NaOH for various time periods. Magnification = 10 µm. Keynotes: (**a**–**i**) refers to 0.5 M-6 h, 0.75 M-6 h, 0.5 M-12 h, 0.75 M-12 h, 0.5 M-24 h, 0.75 M-24 h, 0.5 M-30 h, 0.75 M-30 h, 0.5 M-36 h, 0.75 M-36 h, 0.5 M-42 h, and 0.75 M-42. h denotes gelatin obtained from the camel skin treated with 0.5 or 0.75 M NaOH for 6, 12, 24, 30, 36, and 42 h, respectively.

The microstructure of the camel gelatin gels extracted from the skin pretreated with various concentrations of NaOH is presented in Figure 5. A sponge- or coral-like structure was observed in all the gelatin gels. The camel gelatin gel displayed a more compact and denser network with smaller voids with an increase in pretreatment time. Compared to other gelatin samples, smaller voids with fine and compact network were observed in the gelatin extracted from the skin that had been prior treated with 0.75 M NaOH for 24 h and 0.5 M NaOH for 42 h. Nonetheless, in the gelatin samples extracted at 0.5 M NaOH for 42 h, larger continuous strands that might have provided more resistance in the gel against the applied force were observed. This was well reflected in the higher gel strength of this sample (Table 1). Mad-Ali et al. [10] reported that the gelatin obtained from the goat skin pretreated with 0.5 M NaOH for 4 days were more resistant to the force applied as a result of larger strands, thus providing higher gel strength to the gelatin gel. The gelatin from the skin that was pretreated for a shorter time displayed larger voids in the microstructure, which were also observed in the gelatin reported by [10]. Benjakul et al. [13] reported a link between increases in gel strength and the chain length conformation of proteins in the gel matrix. Interestingly, the gelatin from the skin prior treated with 0.5 and 0.75 M NaOH for 6 h exhibited larger voids (coarser gel structure), which reflected the lower gel strength obtained. It is known that gelatins with large voids and a coarser gel network resulted in lower gel strength and were easy to disrupt [10].

Sae-Leaw et al. [29] observed that gelatin microstructure displaying a coarser network possess a lower degree of the interconnected protein chains compared to the gelatin with a finer connected network, thus resulting in weaker gel strength. A significant property that affects the microstructure of gelatin gels has been reported to be the spread of the α-, β-, and γ-chains [30]. This suggested that the pretreatment and extraction conditions influenced the MW distribution, thus affecting the layout of peptide in the network during gelation. The results obtained in this study agree with the work of [10], where the gel strength of the alkaline-pretreated goat skin was a function of the layout and interaction of the gelatin molecules in the gel matrix.

### 3.8. Gelling and Melting Temperatures

**Figure 6:**
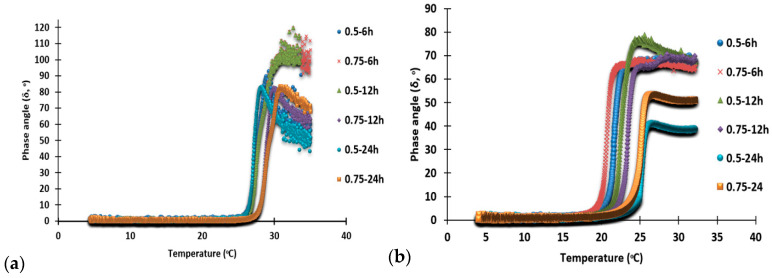
Changes in the phase angle (δ, °) during gelling (**a**) and melting (**b**) process of gelatin solution from camel skin pretreated with 0.5 and 0.75 M NaOH for 6, 12 and 24 h. Ketnotes: 0.5 M-6 h; 0.75 M-6 h; 0.5 M-12 h; 0.75 M-12 h; 0.5 M-24 h; 0.75 M-24 h denotes gelatin obtained from the camel skin treated with 0.5 or 0.75 M NaOH for 6, 12, and 24, respectively.

Gelling and melting temperatures were depicted as a change in the phase angle (δ) of the gelatin solution when cooled from 35 °C to 4 °C and then subsequently heated from 4 °C to 35 °C, as depicted in Figure 6a,b, respectively.

All the camel gelatin samples demonstrated gel formation in the temperature range of 25.8 °C to 20.9 °C when the skin was pretreated at different concentrations of NaOH and time. Other than the changes caused by the NaOH and pretreatment time of the skin, no significant differences were observed in the gelling and melting temperatures for all the samples. Mad-Ali et al., Karnjanapratum et al., and Aksun Tumerkan et al. [10,14,15] recorded similar gelling temperatures for the gelatin derived from goat skin, Asian bull frog (*Rana tigerina*), and frog (23.02 °C to 24.16 °C, 24.05 °C to 24.87 °C, and 28 °C). The gelling temperature of the camel skin gelatin reported in this study was higher than that of the camel skin gelatin obtained from 2.5-, 4.5-, and 7-year-old camels, which reported very low gelling temperatures of 15.2 °C, 11.6 °C, and 11.1 °C [6]. This difference in gelling temperature might be due to the differences in the pretreatments of the skin and the method of extraction of the gelatin. Moreover, the camel gelatin in this study exhibited a higher gelling temperature compared to the gelatin from different sources, such as silver carp (18.7 °C) [12] and bovine gelatin (21.7 °C) [4]. Therefore, the camel gelatin possessed a gelling temperature that ranged between 20.9 °C and 25.8 °C and formed a gel at temperatures close to room temperature. Gelling and melting temperatures are reported to be dependent on the alpha-, β-, and γ-chains content of the gelatin, maturation period of the gel, and temperature [31].

The melting temperature of the gelatin extracted from the pretreated camel skin was in the range of 27.3 °C to 30.5 °C, with different concentrations of NaOH and time. This was found to be higher than the melting temperature of the gelatin from 2.5-, 4.5-, and 7-year-old camel skins (21.6 °C, 18.7 °C, and 18.4 °C, respectively) reported by [6]. It was, however, lower than that of the gelatin from frog skin (43 °C) [15], Asian bullfrog (*Rana tigerina*) (32.2 °C to 34.7 °C) [14], and goat skin (33.07 °C to 34.51 °C) [10]. The melting temperatures (27.34 °C to 30.49 °C) of the camel skin gelatin, and most of the previously reported gelatins from different sources, were always higher than the gelling temperature (25.8 °C to 20.9 °C) because of the energy absorbed by the gelatin gels when melting. If a higher melting temperature in the gel phase of the gelatin could be retained for a longer time, it would confer an advantage of improved mouthfeel to the food products. The different living conditions and variations in temperature for the different species used as the starting material for gelatin are some of the factors responsible for the differences found in the gelling and melting temperatures. Another major factor is the age of the animal, which was related to the cross-link formation. Overall, the camel skin gelatin in this study displayed considerably higher gelling and melting temperatures, which were close to room temperature.

Techno-functional properties of camel skin gelatin (solubility, emulsifying, and foaming properties), as well as turbidity, were also analyzed and have been presented in Supplementary Materials (Figure S2A–D).

## 4. Conclusions

This study explored the optimal conditions required for the pretreatment of camel skin to obtain high-quality gelatin. In-depth characterization revealed that the structural, rheological, and gel-forming properties of gelatin were greatly affected by the pretreatment conditions. This gave a better understanding of the structural and molecular characteristics, responsible for the rheological and physicochemical properties of the gelatin. Overall, the results revealed that the skin pretreated using 0.5–0.75 M NaOH for 42 h demonstrated efficacy for rendering gelatin with high-yield, superior gel strength, and structural features. Camel skin gelatin contained *α*- and *β*- chains as the major components, and these proteins in the gelatin obtained in the preliminary experiment underwent degradation and disappeared from the SDS-PAGE. This study demonstrates that camel skin could be a promising source of halal and BSE-free gelatin. Therefore, future research should focus on improving the color and odor of the gelatin extracted from camel skin.

## Figures and Tables

**Table 1:** Yield and gel strength of camel skin gelatin pretreated with different alkaline concentration for various times.

	Parameters	
Samples	Yield (%)	Gel Strength (g)
0.5 M-6 h	3.2 ± 0.11 ^g^	175.0 ± 11.31 ^f^
0.75 M-6 h	4.2 ± 0.10 ^g^	196.5 ± 14.14 ^ef^
0.5 M-12 h	3.2 ± 0.55 ^g^	200.8 ± 13.08 ^e^
0.75 M-12 h	4.1 ± 0.88 ^g^	247.8 ± 20.15 ^d^
0.5 M-24 h	6.6 ± 0.26 ^ef^	247.0 ± 12.72 ^d^
0.75 M-24 h	8.2 ± 0.44 ^d^	322.8 ± 29.69 ^b^
0.5 M-30 h	5.9 ± 0.66 ^f^	268.8 ± 15.20 ^c^
0.75 M-30 h	7.8 ± 0.15 ^de^	306.8 ± 13.08 ^b^
0.5 M-36 h	9.4 ± 1.22 ^d^	254.3 ± 13.08 ^d^
0.75 M-36 h	16.1 ± 0.89 ^c^	319.5 ± 18.38 ^b^
0.5 M-42 h	18.9 ± 0.76 ^b^	365.5 ± 7.07 ^a^
0.75 M-42 h	22.6 ± 1.65 ^a^	256.3 ± 8.13 ^d^

Keynotes: 0.5 M-6 h: 0.75 M-6 h; 0.5 M-12 h; 0.75 M-12 h; 0.5 M-24 h; 0.75 M-24 h; 0.5 M-30 h; 0.75 M-30 h; 0.5 M-36 h; 0.75 M-36 h; 0.5 M-42 h; 0.75 M-42 h denotes gelatin obtained from the camel skin treated with 0.5 or 0.75 M NaOH for 6, 12, 24, 30, 36, and 42 h, respectively. Different superscripts in the same row indicate significant differences (*p* < 0.05).

## Supplementary Materials

The following are available online at https://www.mdpi.com/article/10.3390/foods10071563/s1, Figure S1: Fourier transformation infrared (FTIR) spectra of gelatin from camel skin pretreated with 0.5 and 1.0 M NaOH for 2 and 4 days; Figure S2: Techno-functional properties (solubility (a), emulsifying activity index (EAI) and emulsifying stability index (ESI) (b), foam expansion (FE) and foam stability (FS) (c), and Turbidity (d) of gelatin solution from camel skin pretreated with 0.5 and 0.75 M NaOH for 6, 12 and 24 h.
