# Supplementary material for: Molecular, Structural, and Rheological Characterization of Camel Skin Gelatin Extracted Using Different Pretreatment Conditions
Source: Foods. 2021 Jul 6;10(7):1563. doi: 10.3390/foods10071563 (PMC8304296; doi:10.3390/foods10071563)
Supplement: Supplementary file 1 [file foods-10-01563-s001.zip › foods-1207093-supplementary.pdf]

Supplemantary Materials

1. Proximate composition

The moisture, ash, fat, and protein contents (with a nitrogen factor of 6.25) of the camel skin were evaluated using the AOAC (2000) methods.

2. Techno-functional properties

2.1. Gelatin solubility determination

Camel skin gelatin solubility (1% solution w/v) was determined using the method described by Kittiphattanabawon et al. (2012) with a slight modification.

2..2. Determination of the emulsifying properties

The method described by Pearce and Kinsella (1978) was used to determine the emulsion activity index (EAI) and emulsion stability index (ESI) of the camel gelatin solution (1% w/v):

Emulsion activity index (EAI) (m^2/g) = $\frac{(2\times 2.303\times abs1)*DF}{(l*v*c)}$,……………………Eq. 1

Emulsion stability index (ESI) (min) = $abs1\times(\frac{\Delta t}{\Delta A})$, …………………………Eq. 2

where **abs1** = absorbance measured immediately after emulsion formation, **abs2** = absorbance measured after 10 min of emulsion formation, **l** = path length of the cuvette, **v** = volume of oil used, **C** = protein content in the aqueous phase (g/mL), **Δt** = 10 min, and **ΔA** = abs1 − abs2.

2.3. Determination of the foaming properties

Foam expansion (FE) and foam stability (FS) of camel gelatin solution (1% w/v) were determined using the method described by Shahidi, Xiao-Qing, and Synowieck (1995). FE and FS were calculated using equations 3 and 4:

FE (%) = $\frac{Vt1}{V0}\times 100$,…………………… Eq. 3

FS (%) = $\frac{Vt2}{V0}\times 100$,…………………… Eq. 4

where $\mathbf{Vt_1}$ = final volume upon whipping, $\mathbf{V_0}$ = initial volume of the gelatin solution, and $\mathbf{Vt_2}$ = volume remaining after 1 h.

2.4. Turbidity

Gelatin samples were prepared to obtain a final concentration of 66.7 g/L. The samples were poured into 1 cm cuvettes at room temperature, and the turbidity was measured using a spectrophotometer (UV-160, Shimadzu, Kyoto, Japan) at 600 nm.

3. Result and discussion

3.1. Proximate analysis of the camel skin

To our knowledge, this study is the first to report the proximate composition and an in-depth characterization of camel skin gelatin. In this study, the raw camel skin contained a moisture content of 68.15%, a protein content of 86.47%, a fat content of 9.19%, and an ash content of 1.04% on dry weigh basis (data not shown). These results suggested that camel skin contained higher amounts of protein and fat contents than other gelatin sources. Ofori (2001) reported that bovine skin contained a crude protein content of 56.46%–69.19% and a fat content of 1.04%–3.58%, which are significantly lower than those of the camel skin. Furthermore, the protein content of the camel gelatin was higher than that of a report by Shyni et al. (2014) for tuna skin (27.7%). However, the higher fat content in the camel skin might become problematic in production as it could lead to reduced clarity of the gelatin and an off-odor. Therefore, further research is required to investigate corrective actions for these issues.

3.2 Solubility

The solubility of the gelatin powder is an important requirement for the efficient demonstration of the other functional properties of gelatin-like emulsifying, foaming, and gelation (Ahmed & Benjakul, 2011). In a colloidal system, the homogenous dispersion of the protein

molecules as a result of the highly soluble proteins assisted in increasing the interfacial properties such as emulsifying and foaming. The solubility of the camel skin gelatins varied depending on the level of alkaline pretreatment applied. As illustrated in **Fig. S2 (A)**, the solubility of the camel gelatin ranged from 86.5% to 97.0%, with pretreatment of 0.75 M NaOH for 24 h displaying the highest solubility. Conversely, pretreatment with 0.5 M NaOH for 24 h exhibited the lowest solubility. These results corroborate with previous reports on gelatin solubility, where fish skin gelatin is known to possess solubility in a range of 89.0% to 96.0% (sea bass skin gelatin) (Sae-Leaw et al., 2016). The gelatin protein is known to contain higher hydrophilic domains at the surface, which could easily interact with the surrounding water molecules to a greater extent, resulting in high solubility (Sae-Leaw et al., 2016). Overall, the high solubility of the gelatin samples is an important functional property and could increase utilization in the food industry as it is a prerequisite for food protein functionality.

3.3. Emulsifying and foaming properties

The surface (interfacial) active properties and the emulsifying and foaming properties of the camel skin gelatin are shown in **Fig. S2 (B & C)**. Significant differences were observed in the EAI among the different gelatin samples and the samples obtained from the skin pretreated with 0.5M/24h and 0.5M/12h, which displayed the highest EAI ($P < 0.05$), and those pretreated with 0.5/0.75M/6h exhibited the lowest EAI. Overall, the EAI of the gelatin increased ($P < 0.05$) as the extraction time increased from 6 to 24 h ($P < 0.05$). The protein denaturation and protein hydrolysis as a result of the longer pretreatment time might have resulted in the protein unfolding and generation of short peptides, which enhanced the migration of proteins to the interface in order to localize and maneuver around the oil droplets. The presence of longer and shorter chain peptides played an important role in displaying efficient emulsion properties. Although the shorter peptides

assisted in forming the emulsion efficiently by quickly migrating to the oil–water interface, the longer peptides assisted in forming thicker and stronger films around the oil droplets, which resulted in an emulsion with higher stability (Kanwate, Ballari, & Kudre, 2019).

The ESI of the gelatin decreased ($p < 0.05$) with an increase in extraction time from 6 to 24 h from an initial value of 4.6 to 3.9 min. The gelatin extracted from camel skins pretreated with 0.5/0.75 M NaOH for 6 h provided a stable and higher ESI (4.5 and 4.6 min, respectively), whereas the gelatin from the camel skin pretreated with 0.5 M NaOH for 24 h exhibited a lower ESI than the other pretreatment conditions. The presence of long-chain peptides in the gelatin prepared under a short time enabled the formation of thicker and stronger films surrounding the oil droplets, which, in turn, stabilized the emulsion system against coalescence or flocculation for a longer time. The high stability of an emulsion is due to a higher content of the intact α- and β-chains with increased hydrophobic interactions with the oil surface (Duan, Zhang, Liu, Cui, & Regenstein (2018).

The foaming properties of the gelatin derived from the camel skin pretreated with different concentrations of NaOH for different time periods are depicted in **Fig. S2 (C)**. The important characteristics of the gelatin solution are the ability to form stable foams. Overall, the foam expansion and foam stabilizing properties of the camel gelatin were similar, with the highest value of 109 (106%) and the lowest value of 106 (103%), respectively. Gelatin from skin pretreated with 0.75 M/0.5 M NaOH for 6 and 12 h gave the highest FE (109%), while gelatin pretreated with 0.75 M NaOH for 12 h exhibited the lowest FE (106%). The film-forming ability at the air–water interface is responsible for the foam formation of proteins. The ability to foam occurs in proteins that easily adsorbed while bubbling on the newly created air–liquid interface and rearranged molecularly at the interface (Damodaran, 1997). The source of gelatin and the extent of the

degradation of the polypeptide chains, intrinsic properties, composition, and conformations of the protein in solution are the factors that influence the foaming properties of gelatin.

The foam stability (FS) of the gelatin decreased ($P < 0.05$) with an increase in the pretreatment time. Camel skins pretreated with 0.75 M NaOH for 6 h had the highest FS, whereas a low FS was recorded in the gelatin from skins pretreated with 0.75 M NaOH for 12 h and 0.5 M NaOH for 24 h. The decrease in FS might be due to the gravitational drainage of water, which caused instability in the foam. Yu and Damodaran (1991) reported that the gravitational drainage of water was a major factor contributing to foam instability. Film thinning and rupture of the gas bubbles have reportedly occurred as a result of liquid drainage from the lamella film when two gas bubbles collide, causing coalescence of the bubbles and loss of stability in the foam (Damodaran, 2005). Larger and longer MW peptides generated as a result of a shorter pretreatment time and could lead to the stabilization of the protein film at the interface more effectively, thus forming stronger films, and greater FS. The results from this study revealed that the interfacial properties of camel skin gelatin were greatly influenced by the alkaline pretreatment and time.

3.4. Turbidity

In food applications where the addition of gelatin is used as a thickening agent, the degree of clarity of a solution (turbidity) is very important. As depicted in **Fig. S2 (D)**, the highest turbidity was recorded in a gelatin solution from pretreated camel skin at 0.75 M NaOH for 24 h. These results agreed with a previous report on gelatin turbidity, where the clown featherback skin gelatin exhibited increased turbidity based on the time of the extraction (Kittiphattanabawon Benjakul, Sinthusamran, & Kishimura, 2016). The increased turbidity of the gelatin solution from the pretreated camel skin at 0.75 M NaOH for 24 h was concurrent with the high gel strength.

Literature reveals that random aggregation of the protein becomes more obvious in gelatin extracted for a longer time and leads to an increase in turbidity (Kittiphattanabawon et al., 2010). Moreover, the degree of clarity in the gelatin solution was directly linked to the efficiency of the filtration process during the gelatin extraction (Muyonga et al., 2004). Future research into defatting of the camel skin as well as bleaching is expected to decrease the turbidity of the camel gelatin solution.

Supplementary Figures

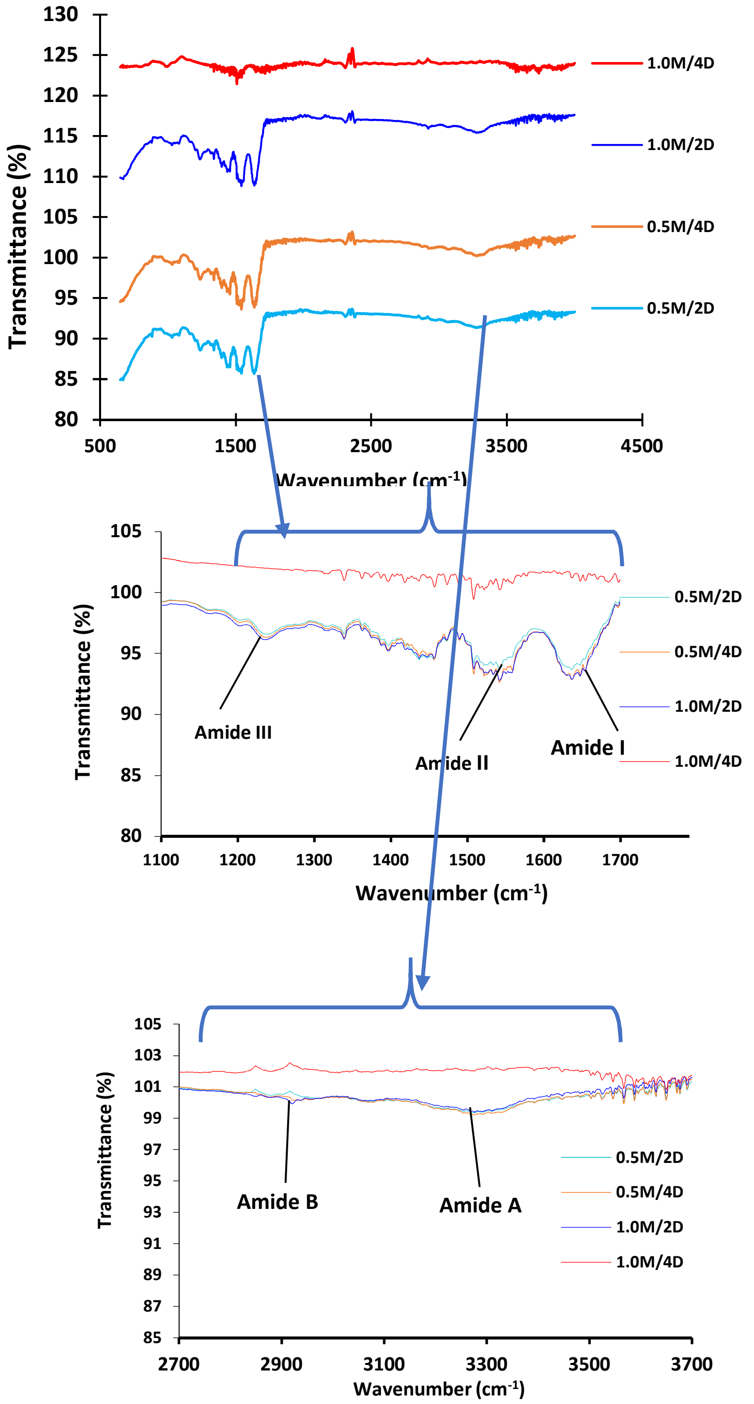

Figure S1. Fourier transform infrared (FTIR) spectra of gelatin from camel skin pretreated with 0.5 and 1.0M NaOH for 2 and 4 days

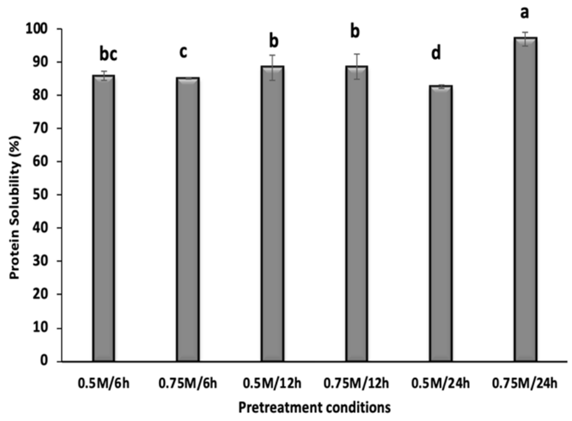

a)

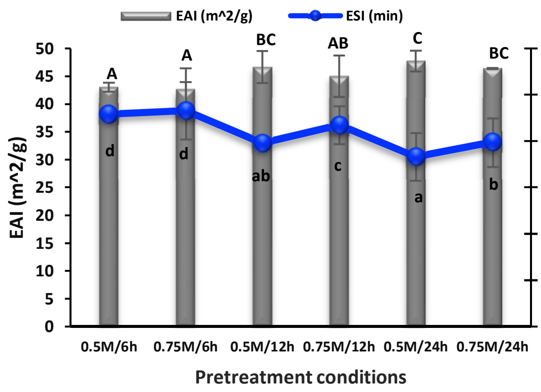

b)

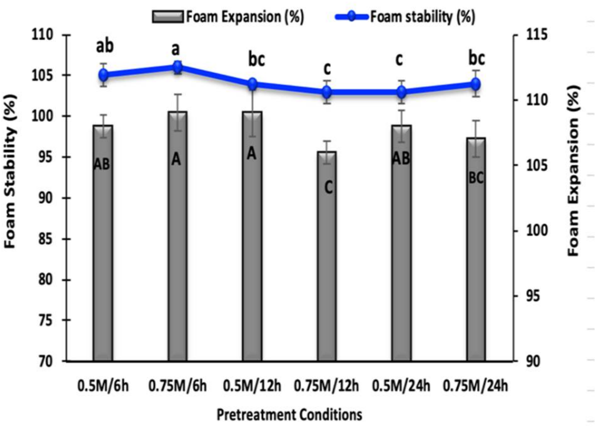

c)

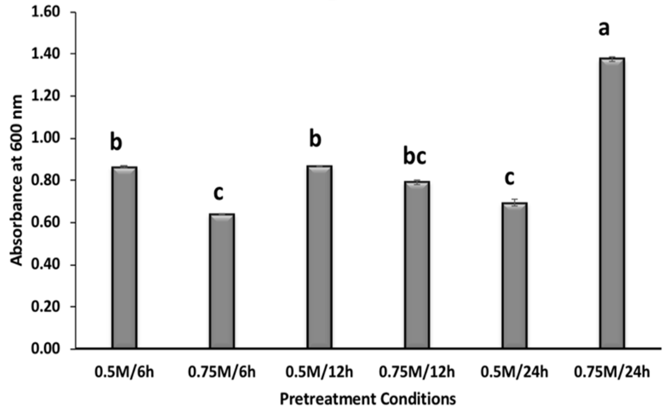

d)

Figure S2. Techno-functional properties (solubility (a), Emulsifying activity index (EAI) and emulsifying stability index (ESI) (B), Foam expansion (FE) and foam stability (FS) (C) and Turbidity (D) of gelatin solution from camel skin pretreated with 0.5 and 0.75M NaOH for 6, 12 and 24h.

References

1. Ahmed, M., & Benjakul, S. (2011). Characteristics of gelatin from the skin of unicorn leather jacket as influenced by acid pre-treatment and extraction time. *Food Hydrocolloids, 25,* 381-388.
2. Damodaran, S. (1997). Protein-stabilized foams and emulsions. In S. Damodaran, & A. Paraf (Eds.), Food proteins and their applications (pp. 57-110). New York: Marcel Dekker Inc
3. Damodaran, S. (2005). Protein stabilization of emulsions and foams. Journal of Food Science, 70: R54-R65.
4. Duan, R., Zhang, J., Liu, L., Cui, W. & Regenstein, J. M. (2018). The functional properties and application of gelatin derived from the skin of channel catfish (Ictahrus punctatus). *Food Chemistry*, *239,* 464-469.
5. Kanwate, B. W., Ballari, R. V., & Kudre, T. G. (2019). Influence of spray-drying, freeze-drying and vacuum-drying on physicochemical and functional properties of gelatin from Labeo rohita swim bladder. *International journal of Biological Macromolecules*, *121*, 135-141.
6. Kittiphattanabawon, P., Benjakul, S., Visessanguan, W., & Shahidi, F. (2010). Comparative study on characteristics of gelatin from the skins of brown banded bamboo shark and blacktip shark as affected by extraction conditions. *Food Hydrocolloids, 24*, 164-171.
7. Kittiphattanabawon, P., Benjakul, S., Visessanguan, W., & Shahidi, F. (2012). Effect of extraction temperature on functional properties and antioxidative activities of gelatin from shark skin. *Food and Bioprocess Technology*, *5*(7), 2646-2654.

8. Kittiphattanabawon, P., Benjakul, S., Sinthusamran, S., & Kishimura, H. (2016). Gelatin from clown featherback skin: Extraction conditions. *LWT, 66*, 186-192

9. Muyonga, J. H., Cole, C. G. B., & Duodu, K. G. (2004). Fourier transform infrared (FTIR) spectroscopic study of acid soluble collagen and gelatin from skins and bones of young and adult Nile perch (*Lates niloticus*). *Food Chemistry, 86,* 325-333.

10. Ofori, S.K., (2001). Effects of processing methods and storage conditions on the microflora and chemical content of cattle skin „wele". A dissertation submitted to School of Graduate Studies, KNUST. Pp. 40-50.

11. Pearce, K. N., & Kinsella, J. E. (1978). Emulsifying properties of proteins: evaluation of a turbidimetric technique. *Journal of Agricultural and Food Chemistry, 26,* 716-723.

12. Sae-Leaw, T., Benjakul, S & O'brien, N.M. (2016). Effect of pretreatments and defatting of seabass skins on properties and fishy odor of gelatin. Journal of Food Biochemistry, 40 (6), 741-753.

13. Shahidi, F., Xiao-Qing, H., & Synowiecki, J. (1995). Production and characteristics of protein hydrolysates from capelin (*Mallotus Villosus*). *Food Chemistry, 53,* 285-293.

14. Shyni, K., Hema, G. S., Ninan, G., Mathew, S., Joshy, C. G., & Lakshmanan, P. T. (2014). Isolation and characterization of gelatin from the skins of skipjack tuna (*katsuwonus pelamis*), dog shark (*scoliodon sorrakowah*), and rohu (*labeo rohita*). *Food Hydrocolloids*, *39*, 68-76.

15. Yu, M., A., & Damodaran, S. (1991). Kinetics of protein foam destabilization: evaluation of a method using bovine serum albumin. *Journal of Agriculture and Food Chemistry,* 39:1555-1562.
